# Identification of a putative polyketide synthase gene involved in usnic acid biosynthesis in the lichen *Nephromopsis pallescens*

**DOI:** 10.1371/journal.pone.0199110

**Published:** 2018-07-18

**Authors:** Yi Wang, Changan Geng, Xiaolong Yuan, Mei Hua, Fenghua Tian, Changtian Li

**Affiliations:** 1 Key Laboratory of Forest Plant Cultivation and Utilization, Yunnan Academy of Forestry, Kunming Yunnan, China; 2 Engineering Research Center of Chinese Ministry of Education for Edible and Medicinal Fungi, Jilin Agricultural University, Changchun, China; 3 State Key Laboratory of Phytochemistry and Plant Resources in West China, Kunming Institute of Botany, Chinese Academy of Sciences, Kunming, China; Universite Paris-Sud, FRANCE

## Abstract

Usnic acid is a unique polyketide produced by lichens. To characterize usnic acid biosynthesis, the transcriptome of the usnic-acid-producing lichen-forming fungus *Nephromopsis pallescens* was sequenced using Illumina NextSeq technology. Seven complete non-reducing polyketide synthase genes and nine highly-reducing polyketide synthase genes were obtained through transcriptome analysis. Gene expression results obtained by qPCR and usnic acid detection with LCMS-IT-TOF showed that *Nppks7* is probably involved in usnic acid biosynthesis in *N*. *pallescens*. Nppks7 is a non-reducing polyketide synthase with a MeT domain that also possesses beta-ketoacyl-ACP synthase, acyl transferase, product template, acyl carrier protein, C-methyltransferase, and Claisen cyclase domains. Phylogenetic analysis shows that Nppks7and other polyketide synthases from lichens form a unique monophyletic clade. Taken together, our data indicate that *Nppks7* is a novel *PKS* in *N*. *pallescens* that is likely involved in usnic acid biosynthesis.

## Introduction

Usnic acid is a unique natural compound produced by lichens that has antibacterial [1], antiviral[2], and antitumor[3,4] bioactive properties. Several reviews of the bioactive properties of usnic acid have highlighted its pharmaceutical value [5–8]. Chemoenzymatic synthesis analysis has demonstrated that methylphloracetophenone is the precursor of usnic acid [9], and it was hypothesized that usnic acid biosynthesis is associated with non-reducing polyketide synthase (PKS) with a C-methyltransferase (MeT) domain. However, the biosynthesis of usnic acid remains unclear. As in the analysis of other lichen metabolites, there are several challenges limiting the application of techniques typically applied to other organisms, such as gene knockout or heterologous expression, to revealing mechanisms of lichen metabolite biosynthesis [10]. Lichens are stable, self-supporting symbioses between fungi (lichen-forming fungi) and photoautotrophic algal partners [11]. However, lichen-forming fungi have been demonstrated to synthesize usnic acid and many other interesting bioactive substances present in lichens, rather than the algal partner [12]. Consequently, many lichen-forming fungi have been isolated, but under laboratory conditions, these lichen-forming fungi have failed to produce bioactive compounds detected in the lichen thallus [10,13]. Further, there is no universal and effective transformation method for lichen-forming fungi [14,15].

Although several PKS genes have been cloned from lichen-forming fungi [16–18], functional characterizations of PKS have been limited to bioinformatics approaches. Some researchers have attempted the heterologous expression of lichen PKS [16,19,20], but were unable to demonstrate *de novo* biosynthesis of a lichen metabolite. However, two research teams have used qRT-PCR and HPLC techniques to show that putative PKS-related genes are associated with the biosynthesis of target lichen metabolites [10,21].

Although most lichen-forming fungi in the absence of their respective photobionts exhibit a profile of natural products that differs from profiles occurring in wild symbionts, a few lichen-forming fungi can produce usnic acid in laboratory conditions, such as *Neuropogon sphacelatus* in Luria-Bertani (LB) medium [22] and *Nephromopsis pallescens* in malt-yeast (MY) medium [23]. Additionally, extracts of the lichen-forming fungus *N*. *pallescens* have exhibited antifungal activity [24] and anti-*Helicobacter pylori* bioactivity [23]. In this study, transcriptomic data from the usnic-acid-producing lichen-forming *N*. *pallescens* fungus was obtained by RNA-Seq. Putative PKS genes involved in usnic acid biosynthesis obtained by bioinformatic analysis were then confirmed using a combined qRT-PCR and LCMS-IT-TOF analysis.

## Materials and methods

### Lichen-forming fungi

Lichen-forming fungi *N*. *pallescens* (KOLRI-040516) was acquired by Jae-Seoun Hur from the Korean Lichen Research Institute (KoLRI), Sunchon National University. The lichen-forming fungus was isolated by the spore discharge method, and its identity was then confirmed by ITS sequence analysis [24].

### Transcriptome sequence and LCMS analysis

Mycelia of the lichen-forming fungi *N*. *pallescens* were cultured in 100 mL of 1.5% malt-yeast (MY) liquid medium (Difco, Lawrence, Kansas, USA) in 250-mL triangular flasks at 15°C with shaking at 100 rpm. After 2 months of culturing, the mycelia were sampled, immediately submerged in liquid nitrogen, and preserved for RNA extraction. Other culture medium was collected by filtration and extracted using 100 mL of ethyl acetate. The extract was then evaporated with a Buchi Rotavapor (Flawil, Switzerland). The crude extracts were redissolved in 2 mL of methanol (Merck, Darmstadt, Germany), after centrifugation at 12,000 × *g* and filtration through a 0.22-μm filter membrane. LCMS analyses were performed on the LCMS-IT-TOF system (Shimadzu, Kyoto, Japan) with an Agilent Eclipse Plus C18 column (100 × 2.1 mm i.d., 1.8 μm, Agilent Technologies) at 30°C. The A and B mobile phases for LCMS analysis were water with 0.05% formic acid (Aladdin Chemistry Co., Ltd., Shanghai, China) and acetonitrile (Merck) with 0.05% formic acid, respectively. The flow rate was 0.2 mL/min. A binary gradient elution was performed as follows: a 5–100% linear gradient of B for 12 min; maintained with 100% B for 4 min; quickly returned to the initial 5% B for 2 min. The injection volume was 2 μL for each LCMS analysis. The mass resolution was approximately 10,000 full width at half maximum. Accurate masses were corrected by calibration using sodium trifluoroacetate clusters. MS experiments were conducted using an automatic pattern in both positive and negative ion modes. Analytical conditions were as follows: spray voltage, 4.50 kV or -3.50 kV; detector voltage, 1.65 kV; drying gas pressure, 110.0 kPa; nebulizing gas (N2) flow, 1.5 L/min; curved desolvation line temperature, 200°C; heat block temperature, 200°C; scan range of 100–1000 m/z for MS. The Shimadzu Composition Formula Predictor was used to infer the molecular formula. The detection of usnic acid (Sigma-Aldrich, St. Louis, MO, USA) was confirmed by comparison to the reference sample with the same retention time (tR = 12.0 min) and mass spectra (positive, m/z 345.0944, [M+H]+, -2.5 mD; negative, m/z 343.0812, [M‒H]-, -1.1 mD; S1 Fig) and UV spectrum (S2 Fig). For lichen-forming fungi samples in which usnic acid production was confirmed through LCMS-IT-TOF analyses, the frozen mycelia were ground into powder for RNA extraction.

Total RNA was isolated using the RNeasy Plant mini Kit (Qiagen, Hilden, Germany). The concentration and quality was examined using a NanoDrop 2000 spectrophotometer (Thermo Scientific, Hudson, NH, USA) and 0.8% agrose gel electrophoresis. The construction of the libraries and RNA-Seq protocol were performed by the Personal Biotechnology Co., Ltd (Shanghai, China). The TruSeq RNA Sample prep Kit (Illumina, San Diego, CA, USA) was used for purification and fragmentation of mRNA. First strand cDNAs were synthesized by reverse transcriptase using the cleaved RNA as a template, and second-strand cDNAs were generated by DNA polymerase I. The ends of DNA fragments were modified and ligated with adapters, and the cleaned ligation products were used as templates for PCR to enrich the products. The cDNA library was obtained, and the quality was examined with a PicoGreen assay kit for the Agilent 2100 Bioanalyzer (Santa Clara, CA, USA). The constructed cDNA libraries were sequenced on the Illumina NextSeq 2500 platform by Shanghai Personal Biotechnology Co., Ltd.

Following sequencing of the cDNA library, high-quality clean reads were generated by trimming the raw reads to remove adapter sequences, low-quality reads with Q values <20 and ambiguous bases (‘N’). The clean reads were then assembled de novo using the Trinity platform (http://trinityrnaseq.sf.net.). After Inchworm, Chrysalis, and Butterfly software analyses, the finished transcript was obtained [25]. Unigenes were obtained by clustering the top-hits from BLASTX searches of the transcript. Both evolutionary genealogy of genes: non-supervised orthologous groups (eggNOG) enrichment analysis and Kyoto Encyclopedia of Genes and Genomes (KEGG) pathway analysis were performed to predict the underlying functions of unigenes. We used the hypergeometric test to examine terms and used Bonferroni correction to adjust *p*-values for multiple comparisons [26]. To seek putative *PKS* genes, BLAST searches with conserved KS domains were made to unigene databases from the finished transcriptome. After BLAST searches, putative unigenes with KS domains were obtained. To obtain the open reading frames (ORFs) of unigenes, the putative unigenes were analyzed with NCBI ORFfinder and FGENESH software. The deduced unigene proteins were analyzed with domain analysis software (http://nrps.igs.umaryland.edu/index.html and https://www.ncbi.nlm.nih.gov/Structure/cdd/wrpsb.cgi). The putative PKSs with complete ORFs were selected based on the length and domain organization of putative unigenes.

### *PKS* cloning

Through transcriptome analysis, the sequences of seven complete non-reducing *PKS* genes from *N*. *pallescens* were obtained. Specific primers for gene cloning were designed base on the transcriptome sequences of the seven *PKS* genes (S1 Table). The RNeasy Plant Mini Kit (Qiagen) was used to isolate RNA from *N*. *pallescens*. The Super Script II reverse transcriptase (Invitrogen, Carlsbad, CA, USA) was used to generate cDNA. The full *PKS* gene from *N*. *pallescens* was cloned into the pUC19 vector using cDNA as a template and the pEASY-Uni Seamless Cloning and Assembly Kits (TransGen, Beijing, China).

### Phylogenetic analyses

Seven non-reducing PKS protein sequences that were deduced from cDNA sequences were used in BLAST searches, respectively. The three most similar sequences and sequences with known functions were used in phylogenetic analyses. A total of 40 fungal PKS sequences were retrieved from GenBank to analyze the relationships between PKS sequences obtained from *N*. *pallescens* and known fungal PKS sequences (S2 Table). These PKS protein sequences were aligned using Clustal W as implemented in MEGA 7.0.14 [27]. Phylogenetic trees were constructed using the minimum evolution method in MEGA 7.0.14 with 1000 bootstrap replicates.

#### Different cultures of lichen-forming fungi *N*. *pallescens* and HPLC-MS analysis

In a previous study [23], we found that media composition influenced the production of antibacterial compounds by *N*. *pallescens*. Therefore, *N*. *pallescens* mycelia were transferred into 100-mL volumes of various broths including the following: MY (1.5% malt-yeast, Difco); MYM (MY+2% mannitol); PDB (2.5% potato dextrose broth); MS (0.5% Murashige and Skoog medium (Chembase, Shanghai, China); 5 g/L glucose); CMG (10g/L casein peptone, 5g/L maltose, 10g/L glucose); SMG (10g/L soya peptone, 5g/L maltose, 10g/L glucose); and TMG (10g/L tomato extract, 5g/L maltose, 10g/L glucose). Mycelia of the lichen-forming fungus *N*. *pallescens* were cultured in 100 mL of different liquid media in 250-mL triangular flasks at 15°C with shaking at 100 rpm. Three independent biological replicates were cultured. After 2 months of culture without light, the mycelia were harvested by filtration with sterilized Miracloth (Merck Millipore, MA, USA) for q-PCR, and the culture liquid was extracted with 100 mL of ethyl acetate to obtain crude extracts for LCMS-IT-TOF detection, respectively.

#### qPCR detection of expression of non-reducing *PKS*s

The expression of seven putative *PKS* genes in different media were detected by q-PCR with special primers (S3 Table). The primers were designed using Beacon Designer 7.90 software (Premier Biosoft, Palo Alto, CA, USA). RNA from different samples was extracted with the RNeasy Plant Mini Kit (Qiagen). From 1 μg of total RNA, cDNA synthesis was performed with a reverse transcriptase kit (TaKaRa Super RT Kit; TaKaRa, Shiga, Japan) according to the manufacturer's instructions. About 500 ng of cDNA from each of the seven samples was used as the template for each qRT-PCR reaction, and the reaction was conducted using SYBR Green (Invitrogen). The specific cDNAs were amplified in a 25-μL reaction volume. PCR reactions were performed using a PCR thermal cycler (ABI 7300; Applied Biosystems, Foster City, CA, USA) with an annealing temperature of 60°C. To estimate the relative mRNA expression level, β-tubulin was used as the reference gene, and the 2^(-ΔΔC(T))^ method was used to test gene expression levels. At least three independent biological replicates and three technical replicates for each biological replicate were analyzed using q-PCR for each sample to ensure reproducibility and reliability. All the assays were compared using one-way analysis of variance (ANOVA) followed by Tukey’s multiple comparisons test.

## Results

### Transcriptome de novo assembly

**Fig 1:**
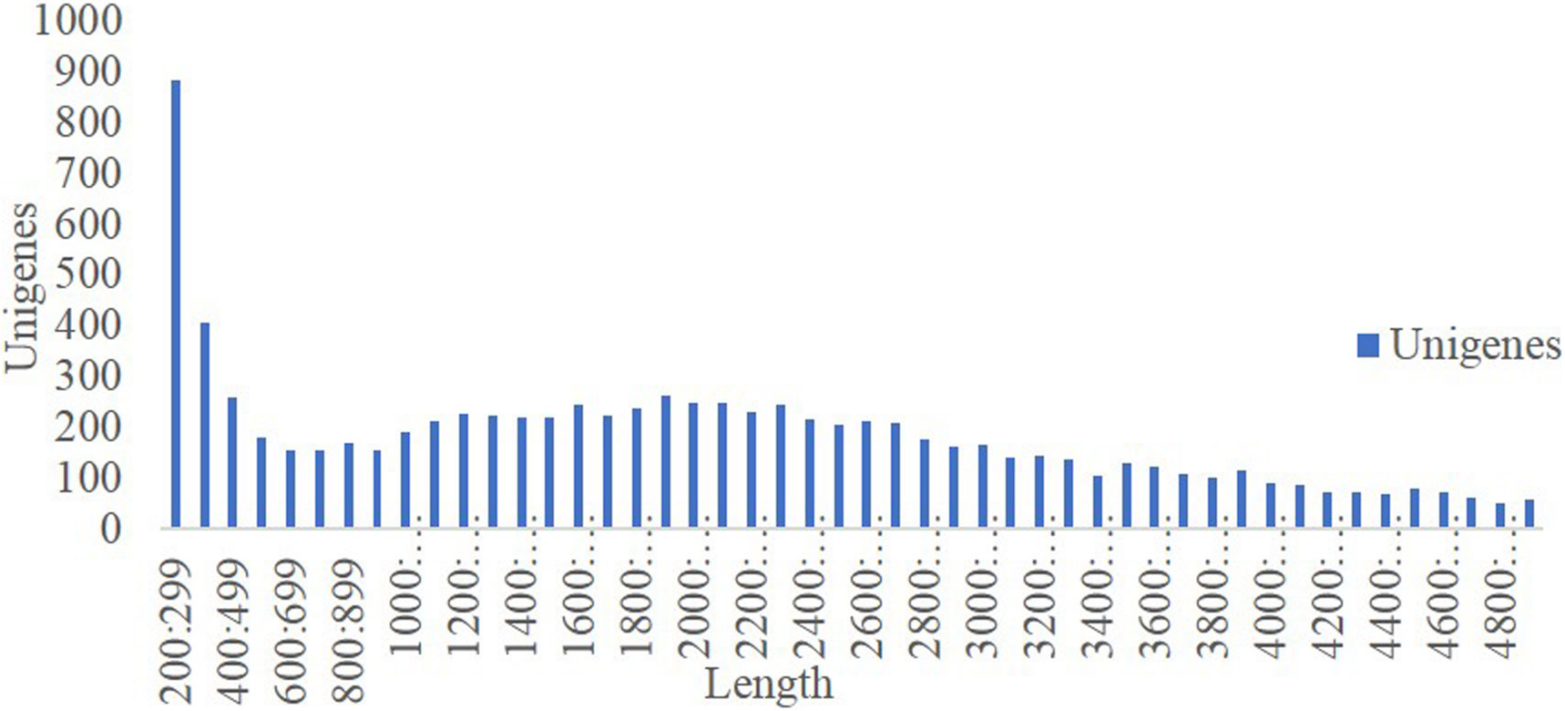
The length distribution of unigenes, the horizontal coordinates are unigene lengths and the vertical coordinates are numbers of unigenes.

To obtain the transcriptome of usnic-acid-producing *N*. *pallescens*, an RNA-Seq library was constructed from mycelia and sequenced using Illumina Next Seq. We obtained 9,683,470,092 bases from 67,422,278 cleaned reads. Ultimately, 9,636 unigenes were identified from assembled transcripts. The mean unigene length was 2419 bp with lengths ranging from 200 to 15,815 bp, and the mean N50 was 3,393 bp (Fig 1). Approximately 75.5% of the unigenes contained more than 1000 bp. The sequence data generated in this study have been deposited into NCBI (accession number SRP091413).

### Functional annotation analysis

**Fig 2:**
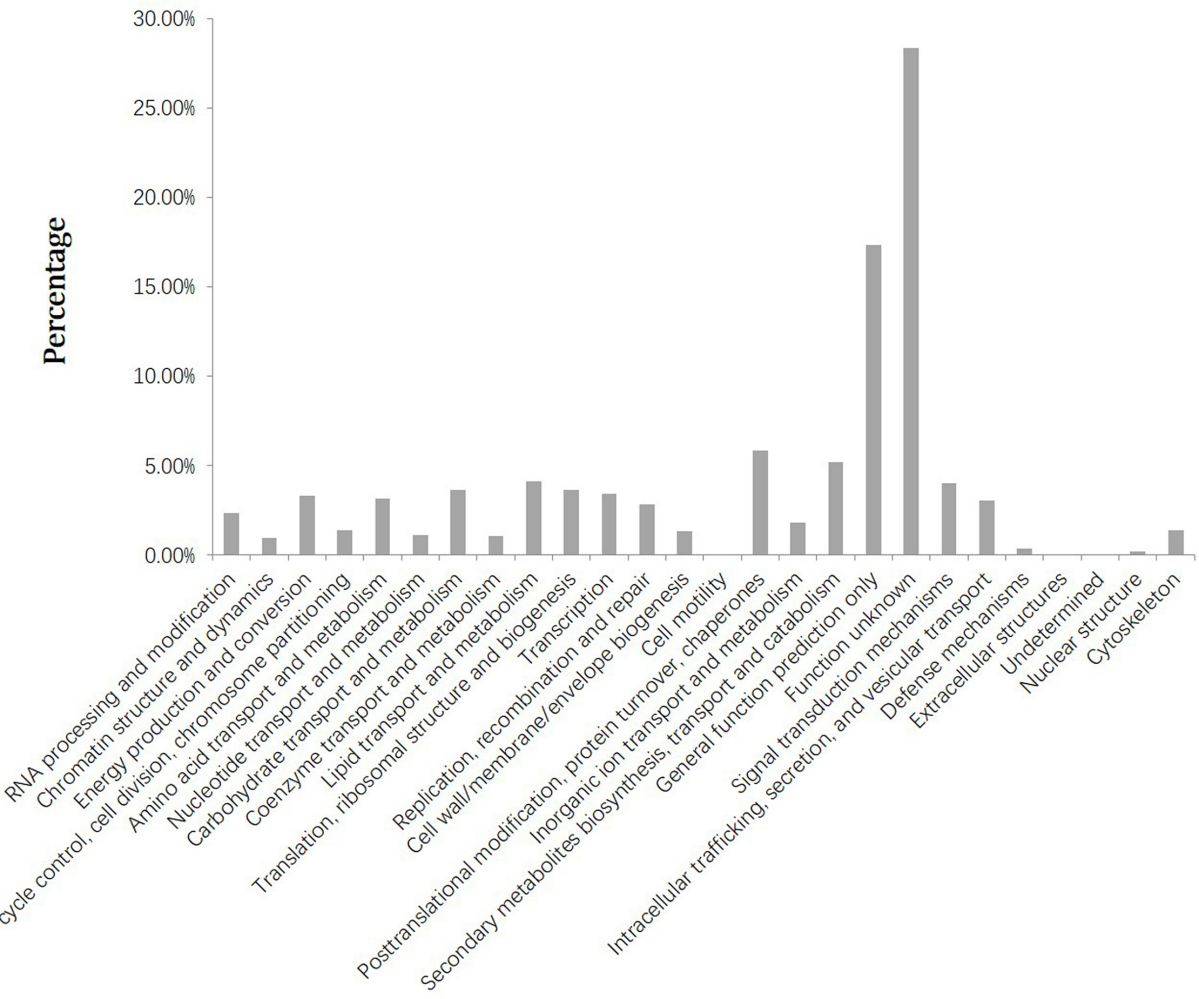
Histogram presentation of eggNOG classification of the assembled unigenes.

Evolutionary genealogy of genes: non-supervised orthologous groups (eggNOG) is a database that provides orthologous groups for 943 Bacteria, 69 Archaea, and 121 Eukaryotes [28]. In this study, 11,729 unigenes were assigned to the eggNOG classification, distributed among 25 eggNOG functional categories (Fig 2). Among these 25 eggNOG categories, “Function unknown” represented the largest group (3,321 unigenes or 28.31%), followed by “General function prediction only” (2,035 unigenes or 17.35%), “Posttranslational modification, protein turnover, chaperones” (685 unigenes or 8.84%), and “Secondary metabolites biosynthesis, transport and catabolism” (608 unigenes or 5.18%).

After unigene annotation and BLASTX search, 99 putative polyketide synthase unigenes were obtained by searching annotation files with polyketide synthases and local BLAST transcriptome assemble files with a beta-ketoacyl-ACP synthase (KS) domains. ORF and domain analyses showed that only 16 unigenes were complete cDNA sequences with a basic polyketide synthase domain (Table 1). The 16 complete *PKS* sequences included seven non-reducing *PKS*s and nine highly-reducing PKSs.

**Table 1:** Complete polyketide synthase was obtained from *N*. *pallescens* transcriptome.

Protein name	Unigene ID	Domain structure
Nppks1	c31_g1_i1	SAT-KS-AT-PT-ACP-TE
Nppks2	c4521_g1_i2	SAT-KS-AT-PT-ACP-ACP-TE
Nppks3	c478_g1_i2	SAT-KS-AT-PT-ACP-ACP-R
Nppks4	c7957_g1_i1	SAT-KS-AT-PT-ACP
Nppks5	c6270_g1_i3	SAT-KS-AT-PT-ACP
Nppks6	c388_g1_i1	SAT-KS-AT-PT-ACP-ACP-MeT-TE
Nppks7	c7776_g1_i11	SAT-KS-AT-PT-ACP-MeT-CLC
Nppks8	c10552_g1_i1	KS-AT-DH-ER-KR-ACP
Nppks9	c7810_g1_i3	KS-AT-DH-MT-ER-KR-ACP
Nppks10	c6216_g1_i3	KS-AT-DH-MT-ER-KR
Nppks11	c7554_g1_i4	KS-AT-DH-MT-KR-ER-KR-ACP
Nppks12	c10551_g1_i1	KS-AT-DH-MT-ER-KR
Nppks13	c5000_g1_i1	KS-AT-DH-ER-KR-ACP
Nppks14	c2844_g1_i1	KS-AT-DH-MT-ER-ACP
Nppks15	c17316_g1_i1	KS-AT-DH-ER-KR-ACP
Nppks16	c7858_g1_i4	KS-AT-DH-KR-ACP

### Gene cloning and domain organization

Seven non-reducing PKS genes were identified from the *N*. *pallescens* transcriptome: *Nppks1* (c31_g1_i1), *Nppks2* (c4521_g1_i2), *Nppks3* (c478_g1_i2), *Nppks4* (c7957_g1_i1), *Nppks5* (c6270_g1_i3), *Nppks6* (c388_g1_i1), and *Nppks7* (c7776_g1_i11). And then seven non-reducing PKS sequences were cloned into the pUC19 vector using the Seamless Cloning and Assembly Kit with special primers. All PKS genes were then confirmed by sequencing. Domain analysis showed that there are two non-reducing PKS sequences including a C-methyltransferase (MeT) domain (Nppks6 and Nppks7). Although both Nppks6 and Nppks7 had the starter unit acyltransferase (SAT), beta-ketoacyl-ACP synthase (KS), acyltransferase (AT), product template (PT), acyl carrier protein (ACP), and C-methyltransferase (MeT) domains, Nppks6 had one more acyl carrier protein domain than Nppks7. Moreover, Nppks7 had a Claisen cyclase (CLC) domain, and Nppks6 had a thioesterase (TE) domain. Two non-reducing PKSs (Nppks5 and Nppks4) possessed the same domain organization (SAT-KS-AT-PT-ACP). Nppks2 and Nppks3 began with similar domain organizations of SAT-KS-AT-PT-ACP-ACP, but their final domains differed, with Nppks2 having a thioesterase (TE) domain and Nppks3 having a reductive releasing domain R. In contrast to Nppks2, Nppks1 had only one acyl carrier protein (ACP) domain, and the domain organization of Nppks1 was SAT-KS-AT-PT-ACP-TE (Table 1).

### Phylogenetic analysis of non-reducing PKS

**Fig 3:**
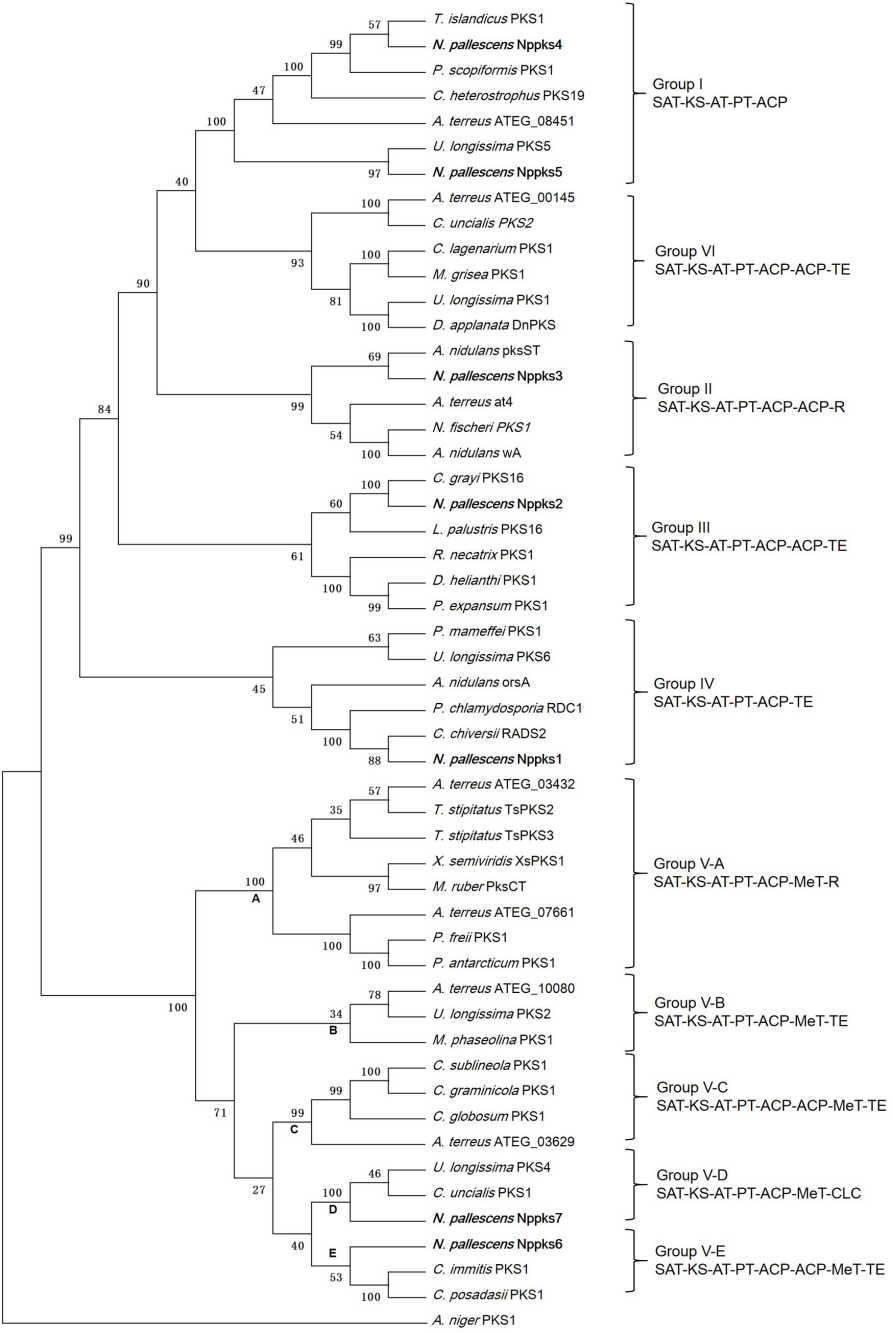
Phylogenetic relationships between *Nephromopsis pallescens* polyketide synthase (PKS) genes and other fungal PKSs. The KS domain of deduced PKS proteins were aligned with fungal PKS sequences retrieved from GenBank. Sequences were aligned using Clustal W and analyzed according to the minimum evolution method. A phylogenetic tree with 1000 bootstrap replicates was generated, with a branch support threshold of 70%. PKSs from the *N*. *pallescens* clade are marked in bold.

The amino acid sequences of the KS domain of seven non-reducing PKSs from *N*. *pallescens* and 41 fungal non-reducing PKSs were used to generate multiple alignments and phylogenetic trees (Fig 3). The branches of the phylogenetic tree can be divided into six main groups according to their domain organization: Group I (SAT-KS-AT-PT-ACP), Group II (SAT-KS-AT-PT-ACP-ACP-R), Group III (SAT-KS-AT-PT-ACP-ACP-TE), Group IV (SAT-KS-AT-PT-ACP-TE), Group V (SAT-KS-AT-PT-ACP-(ACP)-MeT-TE/CLC), and Group VI (SAT-KS-AT-PT-ACP-ACP-TE). The known PKSs in Group I usually were involved in anthraquinone biosynthesis, such as *Aspergillus terreus* ATEG_08451 [29]; Nppks4 and Nppks5 also belonged to Group I. The known PKSs in Group II were usually involved in pigment biosynthesis in fungi; Nppks3 also belonged to Group II. The known PKSs in Group III were also related to fungal pigment biosynthesis and had different cyclization methods relative to Group II. Nppks2 belonged to Group III. The known PKSs in group IV were involved in orsellinic acid biosynthesis, such as orsA from *A*. *nidulans* [30], RADS2 from *Chaetomium chiversii* [31], and RDC1 from *Pochonia chlamydosporia* [32]; Nppks1 belonged to Group IV as well. Group V, consisting of non-reducing PKSs with MeT domains, was complex and varied. For example, it contains five non-reducing PKS with a MeT domain from *A*. *terreus* [29]. Heterologous expression of these five PKS genes from *A*. *terreus* showed that four PKSs with a MeT domain produced different polyketides, while the remaining PKS gene did not produce a detectable product. Phylogenetic analysis showed that Group V can be divided into five sub-groups. Nppks6 and Nppks7 were grouped into their own sub-group. Nppks7, *Cladonia uncialis* PKS1, and *Usnea longissima* PKS4 formed one clade (sub-group D). *Cladonia uncialis* PKS1 was inferred to be involved in usnic acid biosynthesis [10].

### Detection of PKS gene expression and usnic acid

**Fig 4:**
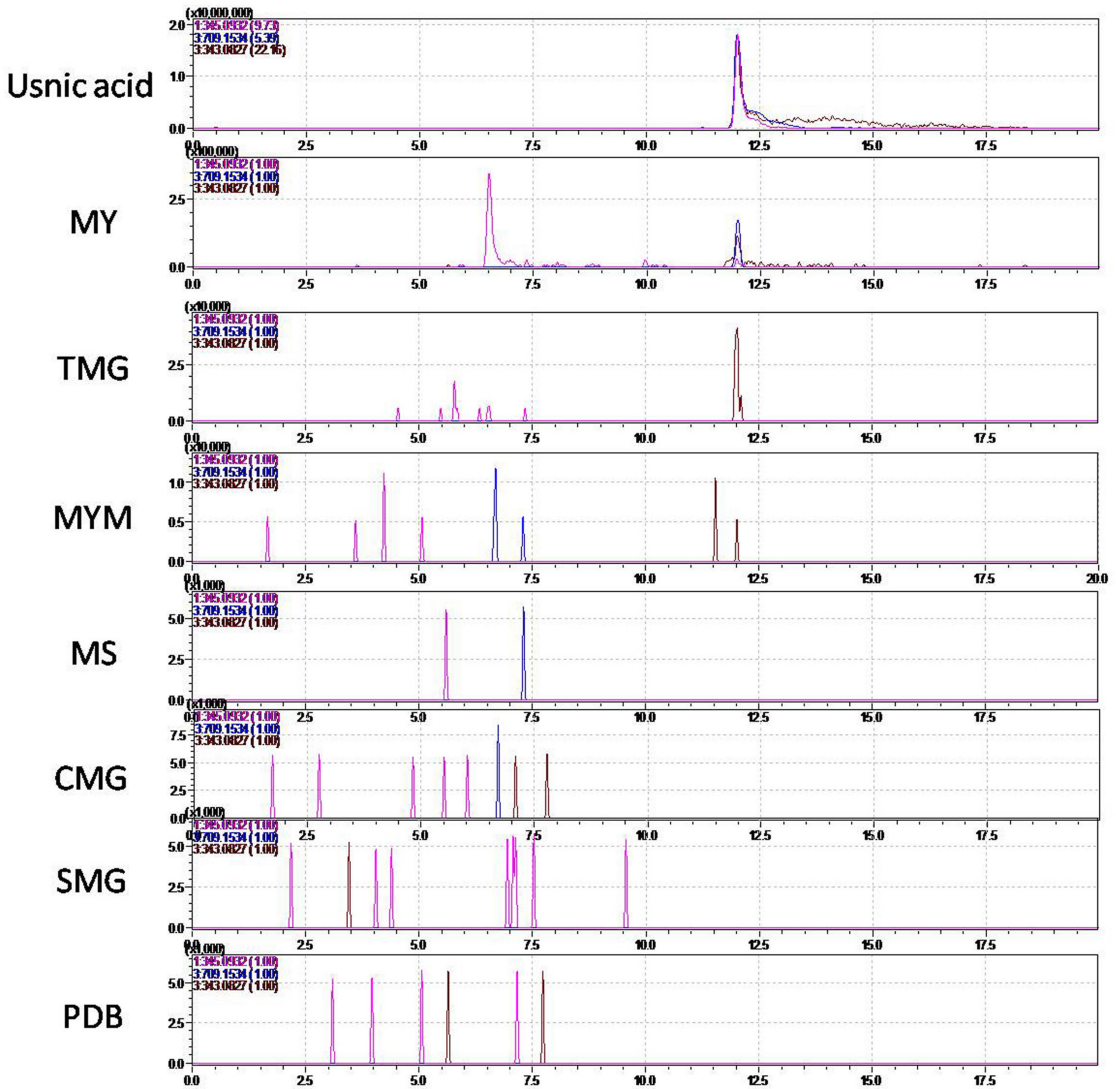
Detection of usnic acid in extracts of different *N*. *pallescens* cultures. Cultures maintained on each of the following media each resulted in different extracts, respectively: MY (1.5% malt-yeast, Difco, Lawrence, USA); MYM (MY+2% mannitol); PDB (2.5% potato dextrose broth, Difco, Lawrence, USA); MS (0.5% Murashige and Skoog medium (Chembase, Shanghai, China); 5 g/L glucose); CMG (10g/L casein peptone; 5g/L maltose, 10g/L glucose); SMG (10g/L soya peptone, 5g/L maltose, 10g/L glucose); and TMG (10g/L tomato extract, 5g/L maltose, 10g/L glucose). The culture media were collected by filtration and extracted with 100 mL ethyl acetate. The crude extracts were redissolved in 2 mL of methanol. The SIM (single ion monitoring) mode of three ions, 345.0932 ([M+H]+), 709.1534 ([2M-H]-), and 343.0827 ([M-H]-) are presented as the main ions in the mass spectra for usnic acid. These three ions were used as monitoring ions in the LCMS analyses.

**Fig 5:**
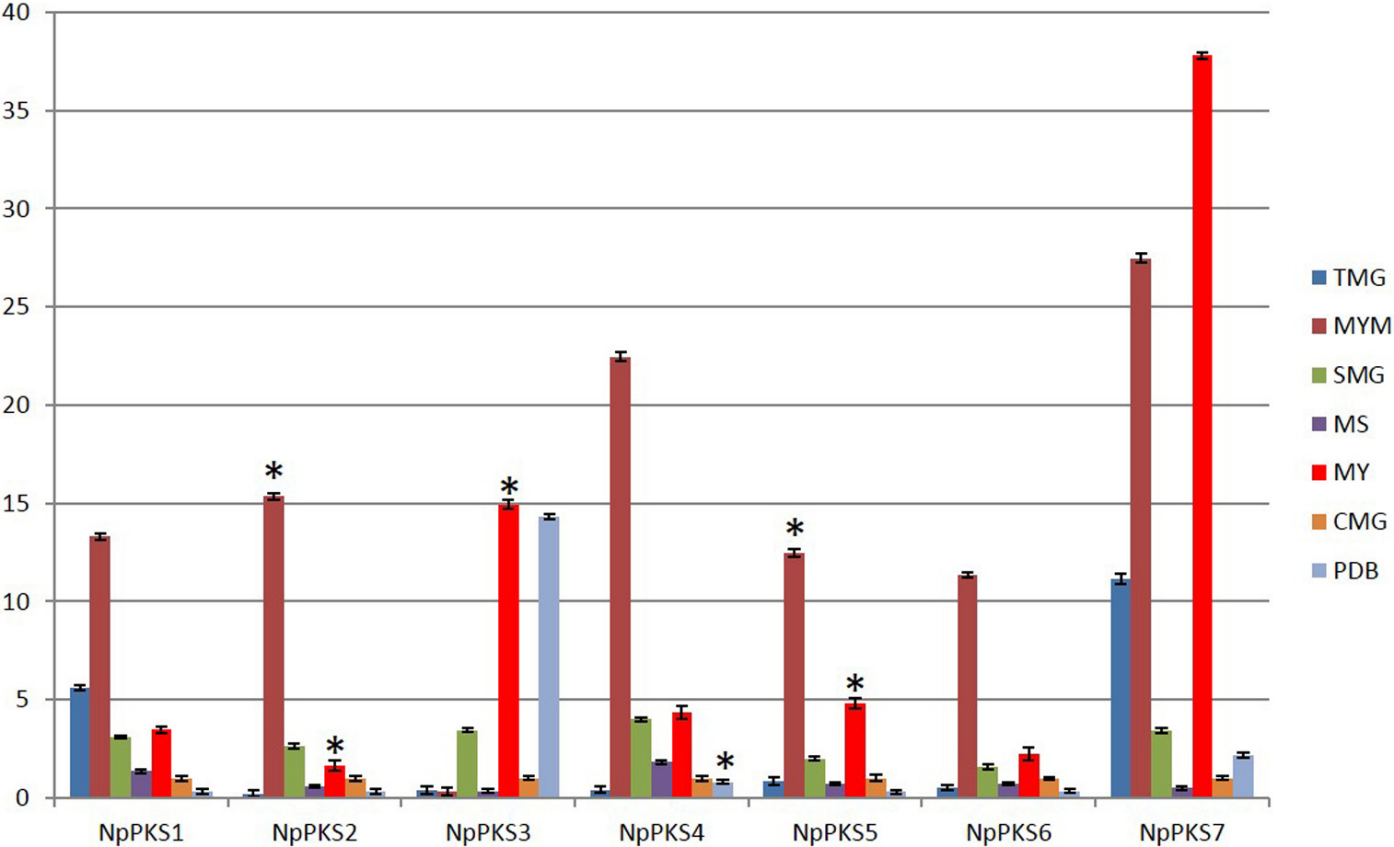
The expression profiles of seven non-reducing PKS genes in seven media. Different cDNA from mycelia were obtained from *N*. *pallescens* cultured in each of the following media: MY (1.5% Malt-Yeast, Difco, Lawrence, USA); MYM (MY+2% Mannitol); PDB (2.5% Potato Dextrose Broth, Difco, Lawrence, USA); MS (0.5% Murashige and Skoog medium (Chembase, Shanghai, China); 5 g/L glucose); CMG (10g/L Casein peptone; 5g/L Maltose, 10g/L Glucose); SMG (10g/L Soya peptone, 5g/L Maltose, 10g/L Glucose); and TMG (10g/L Tomato extract, 5g/L Maltose, 10g/L Glucose). Tubulin was used as the internal reference gene, and all the gene relative expression levels in CMG medium were set to 1. Error bars indicate standard deviations of three biological replicates. Statistically significant differences (*p < 0.01) were determined by an analysis of variation (ANOVA) followed by Tukey's honest significant difference test.

As demonstrated by a previous study, the growth medium influences the production of usnic acid in *N*. *pallescens*. Usnic acid will be produced by the lichen-forming fungi *N*. *pallescens* in MY, MYM, or MYR (MY+2% ribitol) media, but usnic acid was not produced in S2% (Sabouraud+2% glucose), MS, or PDB media [23]. The medium also influences the gene expression of PKS in lichen-forming fungi [17]. Consequently, we incubated the mycelia of *N*. *pallescens* in seven different liquid media, including four different media (i.e., MY, MYM, MS, and PDB) used in a previous study and three media used in this study for the first time with this species. After 2 months of culture, mycelia were harvested for gene expression and usnic acid production analyses. Usnic acid was detected with LCMS-IT-TOF in extracts from MY, MYM, and TMG cultures (Fig 4). According to a previous study [10], the PKS protein associated with usnic acid biosynthesis should be non-reducing. Therefore, we assessed through q-PCR the expression of seven PKS genes in transcriptome data from usnic-acid-producing strains (Fig 5). The q-PCR results show that seven non-reducing PKSs can be highly expressed in MYM medium. *Nppks1* was highly expressed in TMG, and *Nppks3* was highly expressed in MY. However, only *Nppks7* was highly expressed in MY, MYM, and TMG media and weakly expressed in SMG, MS, CMG, and PDB media. Therefore, *Nppks7* appears to be critical for usnic acid biosynthesis in *N*. *pallescens*.

## Discussion

Many clinical drugs, including antibiotics, immunosuppressants, cytotoxins, and cholesterol-lowering substances, are derived from polyketides [33]. Lichens can produce diverse and unique polyketides and thus have high potential pharmaceutical value. However, the slow growth of lichens and lichen-forming fungi limits their direct applications in biotechnology. However, since Miao (2001) proposed harvesting lichen products through genetic approaches [34], several PKS genes have been isolated from lichens and lichen-forming fungi, following the homologous clone method [16,17,35,36]. It is difficult to obtain many genes as well as find new types of genes at one time using homologous cloning methods. Next-generation sequencing (NGS) is a powerful and cost-effective tool that be used to obtain genetic information and large amounts of sequence data quickly [37]. So far, several genomes of lichen-forming fungi have been sequenced on NGS platforms [10,38,39]. Compared to genome sequencing, RNA sequencing (RNA-Seq) is less expensive and easier, and there are more efficient tools to obtain gene expression information from RNA [40]. In this study, RNA sequencing was used to infer the transcriptome of the lichen-forming fungus *N*. *pallescens*, and 16 complete PKS genes were obtained. These sixteen PKSs included nine reducing PKSs and seven non-reducing PKSs. Genome analysis of lichen-forming fungi indicated that lichen-forming fungi have 13–34 complete PKS genes in their genome, including *Cladonia macilenta* with 34 PKS genes, *Cladonia metacorallifera* with 31 PKS genes, *Umbilicaria muehlenbergii* with 20 PKS genes, *Endocarpon pusillum* with 15 PKS genes, *Caloplaca flavorubescens* with 13 PKS genes, and *Cladonia uncialis* with 32 PKS genes [10,38,39]. The different genera of lichen-forming fungi had different PKS gene content in their genomes. Although the mean size of unigenes from transcriptome data was 3393 bp, there are 890 unigenes with lengths over 5000 bp. Although 99 unigenes were annotated as putative polyketide synthases, most unigenes were partial PKS genes. Finally, only 16 compete PKSs were obtained from *N*. *pallescens*, indicating that there are abundant PKS genes in the *N*. *pallescens* genome.

**Fig 6:**
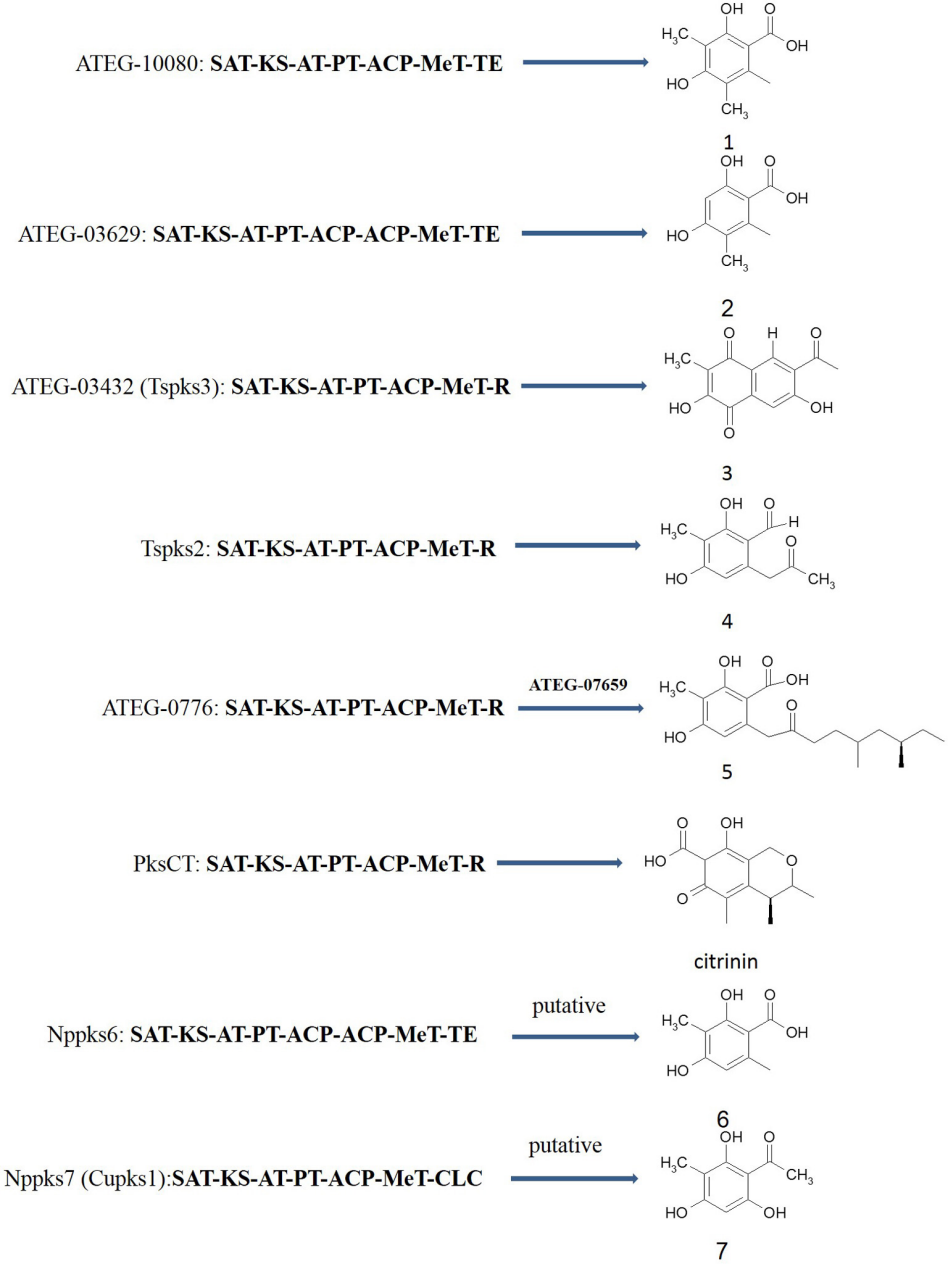
Known non-reducing PKSs with MeT domains and their products. The genes *ATEG-10080*, *ATEG-03629*, *ATEG-03432* and *ATEG-0776* are from *Aspergillus terreus* [29]. Both *Tspks2* and *Tspks3* are from *Talaromyces stipitatus* [43]. *PksCT* is from *Monascus purpureus* [42]. *Cupks1* is from *Cladonia uncialis* [10]. *Nppks6* and *Nppks7* are from *Nephromopsis pallescens*. 1: 3,5-dimethylorsellinic acid; 2: 5-methylorsellinic acid; 3: 6-acetyl-2,7-dihydroxy-3-methylnaphthalene-1,4-dione; 4: 2,4-dihydroxy-3-methyl-6-(2-oxopropyl)benzaldehyde; 5: 2,4-dihydroxy-6-(5,7-dimethyl-2-oxo-trans-3-trans-5-nonadienyl)-3-methylbenzaldehyde; 6: beta-orsellinic acid; 7: methylphloracetophenone. KS, beta-ketoacyl-ACP synthase; AT, acyl transferase; PT, product template domain; ACP, acyl carrier protein; MeT, C-methyltransferase; R, reductive releasing domain; TE, thioesterase domain; CLC, Claisen cyclase domain.

Although many PKS genes were found in lichen-forming fungi, the function of PKSs from lichen-forming fungi is still unclear. The functions are particularly unclear for non-reducing PKSs with a MeT domain (i.e., Group V genes), which are complex and variable. So far, the function of seven non-reducing PKS with MeT domains were confirmed by heterologous expression (Fig 6). It was first confirmed that PksCT with KS-AT-PT-ACP-MeT-R domain is involved in citrinin biosynthesis by heterologous expression of PksCT from *Monascus purpureus* in *Aspergillus oryzae* [41, 42]. Next, five non-reducing PKS genes with MeT domains from *A*. *terreus* were heterologously expressed in the engineered *A*. *nidulans* host [29]. This experiment showed that ATEG-10080, which has a SAT-KS-AT-PT-ACP-MT-TE organizing of domains, is involved in 3,5-dimethylorsellinic acid biosynthesis, ATEG-03629 which has a series of SAT-KS-AT-PT-ACP-ACP-MT-TE domains, is involved in 5-methylorsellinic acid biosynthesis. Although ATEG-10080 and ATEG-03629 had very similar domain organizations, their products differed because ATEG-03629 has one more ACP domain than ATEG-10080. The number of domains will affect the final PKS product, and differences in releasing domains also will lead to variation among their products. ATEG-03432 and ATEG-10080 also possessed a similar domain organization; however, ATEG-10080 has a TE domain, while ATEG-03432 has a R domain. The product of ATEG-03432 was 6-acetyl-2,7-dihydroxy-3-methylnaphthalene-1,4-dione. Hashimoto et al. (2015) reported that the product of TsPKS3, which had the same domain organization as ATEG-03432 from *Talaromyces stipitatus* was 6-acetyl-2,7-dihydroxy-3-methylnaphthalene-1,4-dione, but the product of TsPKS2, which has the same domain organization (i.e., SAT-KS-AT-PT-ACP-MeT-R), is 2,4-dihydroxy-6-(5,7-dimethyl-2-oxo-trans-3-trans-5-nonadienyl)-3-methylbenzaldehyde [43]. According to known non-reducing PKSs with MeT domains, it is clear that the number of domains, and the presence of releasing and PT domains will influence the structure of their product. The phylogenetic analysis showed that PKS sequences with similar domain organizations clustered into the same clade. For example, ATEG-03432, ATEG-07661, TsPKS2, and TsPKS3, which shared the same domain organization (SAT-KS-AT-PT-ACP-MeT-R), were clustered into clade sub-group A. ATEG-10080, which had a similar domain organization (i.e., SAT-KS-AT-PT-ACP-MeT-TE) but a different releasing domain, was clustered into clade sub-group B. Similarly, the PKSs with an additional ACP compared to ATEG-10080, were clustered into sub-group C. The PKS in clade sub-group D bore a SAT-KS-AT-PT-ACP-MeT-CLC domain structure, and the PKS sequences in clade sub-group E bore a SAT-KS-AT-PT-ACP-ACP-MeT-TE domain structure.

The transcriptome of *N*. *pallescens*, which produces usnic acid, showed that there are two non-reducing PKS genes with MeT domains, i.e., Nppks6 and Nppks7. Nppks6 possessed a KS-AT-PT-ACP-ACP-MeT-TE domain organization. Nppks6 and ATEG-03629 had very similar domain organizations. The phylogenetic analysis shows that Nppks6 and ATEG-03629 belonged to different clades. Lichen or lichen-forming fungi can produce large amounts of beta-orsellinic acid, and ATEG-03629 produces 5-methylorsellinic acid [29]. Armaleo et al. (2011) supposed that the PKS gene involved in beta-orsellinic acid biosynthesis needs MeT and TE domains [21]. Therefore, it is possible that Nppks6 is involved in beta-orsellinic acid biosynthesis. Nppks7 possesses a KS-AT-PT-ACP-MeT-CLC domain organization. Compared to known non-reducing PKS sequences, Nppks7 contained an additional Claisen cyclase domain as a releasing domain. Recently, a non-reducing PKS (CuPKS1) from *Cladonia uncialis*, which has a domain structure similar to that of Nppks7, was predicted to be involved in usnic acid biosynthesis based upon genome cluster and gene expression analyses. CuPKS1 was predicted to be involved in methylphloracetophenone biosynthesis and was thus named methylphloracetophenone synthase (MPAS) [10]. Nppks7 and CuPKS1 have the same domain organization (SAT-KS-AT-PT-ACP-MeT-CLC), and the amino acid sequence homology similarity of Nppks7 and CuPKS1 is 81.37%. However, the lichen-forming fungus *C*. *uncialis* in lab culture did not produce usnic acid. Accordingly, the function of CuPKS1 was not confirmed by demonstrating gene expression is associated with usnic acid production. In this study, gene expression by q-PCR detection and usnic acid detection by LCMS-IT-TOF demonstrates that the expression of Nppks7 is consistent with the production of usnic acid in different media. Similarly, the phylogenetic analysis shows that Nppks7, CuPKS1, and UlPKS4 group into a single monophyletic clade. All these results support the conclusion that Nppks7 is involved in usnic acid biosynthesis in *N*. *pallescens* (Fig 6).

## Conclusions

This work presents the first *de novo* transcriptome sequencing analysis of the usnic acid producer *N*. *pallescens*. Sixteen complete PKS gene sequences were obtained from transcriptome data, and seven non-reducing PKS genes were cloned. Our analyses show that it is extremely likely that *Nppks7* is involved in usnic acid biosynthesis. This study provides important information about usnic acid biosynthesis and provides a foundation for obtaining unique lichen products by heterologous expression. This is expected to provide an effective method for the sustainable application of lichen products in the pharmaceutical and perfume industries.

## Supporting information

S1 Table Primers were used in full cDNA clone of PKS from *N*. *pallescens*. (DOCX)

S2 Table The PKS sequence information was used in phylogenetic analysis. (DOCX)

S3 Table Primers were used in detection of PKS gene expression. (DOCX)

S1 Fig The high-resolution mass spectra of usnic acid in both positive (A) and negative modes (B).
(DOCX)

S2 Fig UV spectrum of usnic acid. (DOCX)
