# Supplementary material for: Identification of a putative polyketide synthase gene involved in usnic acid biosynthesis in the lichen Nephromopsis pallescens
Source: PLoS One. 2018 Jul 18;13(7):e0199110. doi: 10.1371/journal.pone.0199110 (PMC6051580; doi:10.1371/journal.pone.0199110)
Supplement: S1 Table — (DOCX) [file pone.0199110.s001.docx]

S1 Table 1 Primers were used in full cDNA clone of PKS from N. pallescens

| Primers name | Sequence (5'-3') *Italic* part showed clone vector sequence | purpose and the length |
| --- | --- | --- |
| NpPKS1WF | *AACAGCTATGACCATGATTACGCC*ATGGCGGATTTGCAGATGTT | 5 party of *NpPKS1,*3024 |
| NpPKS1MR | GCGACAGGACTCCTGCGGCATGTAG |  |
| NpPKS1MF | CTACATGCCGCAGGAGTCCTGTCGC | 3 party of NpPKS1,3249 |
| NpPKS1SR | *ACGACGTTGTAAAACGACGGCCAGT*CTCGAGGCGTTGAAATGATT |  |
| NpPKS2WF | *AACAGCTATGACCATGATTACGCC*ATGGCCACAA CATCCAAGGT | 5 party of NpPKS2,3144 |
| NpPKS2MR | TCTCACAAGACGGCATCTTCTCGGC |  |
| NpPKS2MF | GCCGAGAAGATGCCGTCTTGTGAGA | 3 party of NpPKS2,3230 |
| NpPKS2SR | *ACGACGTTGTAAAACGACGGCCAGT*TCACCAATGTGAGTTTCGCAA |  |
| NpPKS3WF | *AACAGCTATGACCATGATTACGCC*ATGGCGGACCAATTGAAGTT | 5 party of NpPKS3,3504 |
| NpPKS3MR | GGTCGCTACGTGCCACCAAAAGTGC |  |
| NpPKS3MF | GCACTTTTGGTGGCACGTAGCGACC | 3 party of NpPKS3,3291 |
| NpPKS3SR | *ACGACGTTGTAAAACGACGGCCAGT*TTAGTTCAAGAAACCAATCT |  |
| NpPKS4WF | *AACAGCTATGACCATGATTACGCC*ATGATGCCCATCTATACTCC | 5 party of NpPKS4,2424 |
| NpPKS4MR | GCGTCATTTCTAAAGGGATTTGGAG |  |
| NpPKS4MF | CTCCAAATCCCTTTAGAAATGACGC | 3 party of NpPKS4,3030 |
| NpPKS4SR | *ACGACGTTGTAAAACGACGGCCAGT*CTAGCCATAATACTCATCTA |  |
| NpPKS5WF | *AACAGCTATGACCATGATTACGCC*ATGAGTTTCTCAACTACACC | 5 party of NpPKS5,2544 |
| NpPKS5MR | TGATAGGACCTTCTTCAATCGCAAG |  |
| NpPKS5MF | CTTGCGATTGAAGAAGGTCCTATCA | 3 party of NpPKS5,2922 |
| NpPKS5SR | *ACGACGTTGTAAAACGACGGCCAGT*CTAGCTGTAGTACTCTTCCA |  |
| NpPKS6WF | *AACAGCTATGACCATGATTACGCC*ATGGAGCATTTGCTTGAGGA | 5 party of NpPKS6,3025 |
| NpPKS6MR | GACGCAGACGATCATACTGCGGCCT |  |
| NpPKS6MF | AGGCCGCAGTATGATCGTCTGCGTC | 3 party of NpPKS6,2994 |
| NpPKS6SR | *ACGACGTTGTAAAACGACGGCCAGT*TTATGCAAACAATTGAGAATTG |  |
| NpPKS7WF | *AACAGCTATGACCATGATTACGCC*ATGGCTGCTGGTCCTCCTAC | 5 party of NpPKS7,4104 |
| NpPKS7MR | AAGGAAGATAGTCAGAGGAACTTGA |  |
| NpPKS7MF | TCAAGTTCCTCTGACTATCTTCCTT | 3 party of NpPKS7,3741 |
| NpPKS7SR | *ACGACGTTGTAAAACGACGGCCAGT*CTATACAAACCTCGCCAACC |  |
