# Supplementary material for: Identification of a putative polyketide synthase gene involved in usnic acid biosynthesis in the lichen Nephromopsis pallescens
Source: PLoS One. 2018 Jul 18;13(7):e0199110. doi: 10.1371/journal.pone.0199110 (PMC6051580; doi:10.1371/journal.pone.0199110)
Supplement: S2 Table — (DOCX) [file pone.0199110.s002.docx]

S2 Table 2 The PKS sequence information was used in phylogenetic analysis

| PKS name | accession number |
| --- | --- |
| *Arthroderma benhamiae* PKS1 | EFE29950.1 |
| *Aspergillus nidulans* pksST | Q12397 |
| *Aspergillus niger* PKS1 | GAQ46573 |
| *Aspergillus nidulans* ORSA | XP_681178 |
| *Aspergillus nidulans* wA | Q03149 |
| *Aspergillus terreus* at4 | BAB88689 |
| *Aspergillus terreus* ATEG_00145 | EAU38791.1 |
| *Aspergillus terreus* ATEG_03432 | EAU36706.1 |
| *Aspergillus terreus* ATEG_03629 | EAU35431.1 |
| *Aspergillus terreus* ATEG_07661 | EAU31923.1 |
| *Aspergillus terreus* ATEG_08451 | EAU31624.1 |
| *Aspergillus terreus* ATEG_10080 | EAU29529.1 |
| *Chaetomium chiversii* RADS2 | ACM42403.1 |
| *Chaetomium globosum* PKS1 | XP_001228055.1 |
| *Cladonia grayi* PKS16 | ADM79459.1 |
| *Cladonia uncialis* PKS1 | ALA62323.2 |
| *Cladonia uncialis* PKS2 | ANM27730.1 |
| *Coccidioides immitis* PKS1 | XP_001245248.2 |
| *Coccidioides posadasii* PKS1 | XP_003071593.1 |
| *Cochliobolus heterostrophus* PKS19 | AAR90273 |
| *Colletotrichum graminicola* PKS1 | XP_008100672.1 |
| *Colletotrichum lagenarium* PKS1 | BAA18956 |
| *Colletotrichum sublineola* PKS1 | KDN72002.1 |
| *Diaporthe helianthi* PKS1 | OCW33946.1 |
| *Dirinaria applanata* DnPKS1 | ACH72076 |
| *Lepidopterella palustris* PKS16 | OCK77458.1 |
| *Macrophomina phaseolina* PKS1 | EKG10413.1 |
| *Magnaporthe grisea* PKS1 | XP_367294 |
| *Monascus ruber* PksCT | A0A161CEU9.1 |
| *Neosartorya fischeri* PKS1 | XP_001261235.1 |
| *Penicillium antarcticum* PKS1 | OQD84062.1 |
| *Penicillium expansum* PKS1 | XP_016600677.1 |
| *Penicillium freii* PKS1 | KUM65571.1 |
| *Penicillium marneffei* PKS1 | XP_002146110.1 |
| *Phialocephala scopiformis* PKS1 | XP_018066025.1 |
| *Pochonia chlamydosporia* RDC1 | ACD39770.1 |
| *Rosellinia necatrix* PKS1 | GAP86286.1 |
| *Talaromyces islandicus* PKS1 | CRG92129.1 |
| *Talaromyces stipitatus* PKS1 | XP_002339967.1 |
| *Talaromyces stipitatus* Tspks2 | XP_002487778 |
| *Talaromyces stipitatus* Tspks3 | XP_002340070.1 |
| *Usnea longissima* PKS1 | AEM75019 |
| *Usnea longissima* PKS2 | AGI60155.1 |
| *Usnea longissima* PKS4 | AGI60156.1 |
| *Usnea longissima* PKS5 | AGI60157.1 |
| *Usnea longissima* PKS6 | AGI60158.1 |
| *Xanthoparmelia semiviridis* XsePKS1 | ABS58604 |
