# Supplementary material for: Identification of a putative polyketide synthase gene involved in usnic acid biosynthesis in the lichen Nephromopsis pallescens
Source: PLoS One. 2018 Jul 18;13(7):e0199110. doi: 10.1371/journal.pone.0199110 (PMC6051580; doi:10.1371/journal.pone.0199110)
Supplement: S3 Table — (DOCX) [file pone.0199110.s003.docx]

S3 Table 3 Primers were used in detection of PKS gene expression

| Primers name | Sequence (5'-3') | Length of PCR product |
| --- | --- | --- |
| TNpPKS1F | AAGTTGGATGAGAGGATTAG | 78 bp |
| TNpPKS1R | AACAGTGCGTAGATGATT |  |
| TNpPKS2F | TTCGCTGATGACTATGAG | 82 bp |
| TNpPKS2R | GTTCTTCTGGAGTGGTAG |  |
| TNpPKS3F | TGGTCAATCATCGTTCTT | 79 bp |
| TNpPKS3R | AGGTAGCATAGTCTGTTC |  |
| TNpPKS4F | GCCTGGAATGACAAGAACTA | 83 bp |
| TNpPKS4R | TTGTTCATGCGGTGCCCGTG |  |
| TNpPKS5F | AGACTACAGATGATGCTA | 85 bp |
| TNpPKS5R | TTGGTTACTATGAGAATCG |  |
| TNpPKS6F | GTGGATTCGTTGATGATAA | 87 bp |
| TNpPKS6R | GAGTCTATGTCAGTAAGGT |  |
| TNpPKS7F | GGATGACTTGGTAGAATG | 81 bp |
| TNpPKS7R | CTGGATGATATGTATAATGATAG |  |
| tubulinF | ACAGTTCCTACAATGATAGCA | 79 bp |
| tubulinR | CACATTCGTTGGAGGTCTA |  |
