# Supplementary material for: Identification of a putative polyketide synthase gene involved in usnic acid biosynthesis in the lichen Nephromopsis pallescens
Source: PLoS One. 2018 Jul 18;13(7):e0199110. doi: 10.1371/journal.pone.0199110 (PMC6051580; doi:10.1371/journal.pone.0199110)
Supplement: S1 Fig — The high-resolution mass spectra of usnic acid in both positive (A) and negative modes (B). (DOCX) [file pone.0199110.s004.docx]

A

B

S1 Figure 1. The high-resolution mass spectra of usnic acid in both positive (A) and negative modes (B).
