# Supplementary material for: Identification of a putative polyketide synthase gene involved in usnic acid biosynthesis in the lichen Nephromopsis pallescens
Source: PLoS One. 2018 Jul 18;13(7):e0199110. doi: 10.1371/journal.pone.0199110 (PMC6051580; doi:10.1371/journal.pone.0199110)
Supplement: S2 Fig — (DOCX) [file pone.0199110.s005.docx]

S2 Figure 2. UV spectrum of usnic acid.
